# The cost of the perfect body: influence mechanism of internalization of media appearance ideals on eating disorder tendencies in adolescents

**DOI:** 10.1186/s40359-024-01619-7

**Published:** 2024-03-12

**Authors:** Xiaoyan Bi, Qian Liang, Guangyan Jiang, Min Deng, Hongbo Cui, Yankun Ma

**Affiliations:** 1 School of Education, Guangzhou University, NO.230 Waihuan West Road, Panyu District, 510006 Guanzhou, Guangdong Province China; 2 Huizhou Fourth Middle School, NO.4 Xinlian Rode, Huiyang District, 516001 Huizhou, Guangdong Province China; 3 Guizhou University of Finance and Economics, College of Public Management, NO.276 Luchongguan Road, Yunyan District, 550000 Guiyang, Guizhou Province China; 4 School of Humanities and Management, Kunming Medical University, NO.1168 Chunrong West Road, Chenggong District, 650500 Kunming, Yunnan Province China

**Keywords:** Internalization of media appearance ideals, Eating disorder tendenc, Body esteem, Body image disturbance, Social support

## Abstract

**Background:**

Some studies have examined the relationship between internalization of media appearance ideals and eating disorders. However, few have discussed the relationship between eating disorder tendencies. To fill this research gap, this study was to explore the influencing mechanisms of internalization of media appearance ideals on adolescents’ eating disorder tendencies in Chinese cultural context.

**Method:**

The Sociocultural Attitudes Towards Appearance Questionnaire, Eating Attitude Test-26, Physical Self-Description Questionnaire, Body Image Depression Questionnaire and Multidimensional Scale of Perceived Social Support were employed in this study to investigate 1523 adolescents. The collected data were analyzed using SPSS 26.0 and AMOS 24.0.

**Result:**

The results showed that: (1) internalization of media appearance ideals had a significant positive predictive effect on adolescents’ eating disorder tendencies; (2) internalization of media appearance ideals significantly influenced adolescents’ eating disorder tendencies through the mediating role of body esteem and body image disturbance respectively, and also influenced eating disorder tendencies through the chain mediating of both; and (3) social support played a moderating role between body image disturbance and eating disorder tendency.

**Conclusion:**

Our findings suggest distinct pathways through which internalization of media appearance ideals may influence adolescents’ eating disorder tendencies. It is suggested that reducing body image disturbance and enhancing social support can help reducing eating disorder tendency.

## Introduction

In recent years, the prevalence of eating disorders have gradually increased in China [1–3]. Eating disorders are a type of mental disorders characterized by abnormal eating behaviors and psychological disorders, often accompanied by mania, anxiety, suicide and other adverse emotions and behaviors, and it seriously threatens the physical and mental health of individuals [4–6]. The transition from a normal eating state to eating disorders involves a transitional phase that usually includes a series of worse and worse psychological and behavioral changes. It is worth noting that not every individual with eating problems meets the clinical diagnostic criteria [7, 8], and therefore, in addition to screening for patients with clinical eating disorders, researchers have begun to focus on individuals with non-clinical eating disorders, i.e., individuals with eating disorder tendencies. Eating disorder tendencies refers to a range of cognitive, affective, and behavioral tendencies exhibited by individuals with eating as an object, mostly seen in adolescences [9], with characteristics such as high prevalence and universality [10, 11]. The more the eating attitude and behavior deviate from the normal, the more disordered the individual’s eating pattern will be, and the higher the individual’s potential propensity to develop an eating disorders will be [12, 13]. However, there were few researches on the influencing mechanisms of adolescent eating disorder tendencies in China [1, 3, 6, 14–16] Nowadays, media have developed rapidly in China, and have a great impact on the growth of adolescents. Therefore, it is significant for researchers to explore the influencing on eating disorder tendency of Chinese adolescents and prevent and intervene eating disorder. In today’s rapidly developing media, an in-depth investigation into the influencing factors of Chinese adolescents eating disorder tendencies are of great practical significance for the prevention and intervention of eating disorder problems.

### Internalization of media appearance ideals and eating disorder tendencies

Internalization of the media appearance ideals (IMAI) are considered a key risky factor for the development of eating disorders [17–19]. Social comparison and social learning theories suggest that individuals, to some extent, cognitively accept society narrow standard of attractiveness as their own personal standard and will take action to help themselves meet that standard [20, 21]. The implicit process is that disordered eating and extreme exercise are actions taken to internalize an ideal characterized by thinness or muscularity, which is acquired through exposing to supportive social resources. One of the most common kinds of exposure is the portrayal of thinness or muscularity as personal attractiveness or success in the mass media [22], from which individuals learn to conform to and internalize current aesthetic trends by comparing themselves with the images presented in the media. The studies have shown that media (e.g., television, advertisements, and magazines) influence individuals’ food choices and eating behaviors through internalization of media appearance ideals. For example, Couture et al. [23] and Barney et al. [24] found that media internalization of media appearance ideals led individuals to increase body image anxiety and dissatisfaction, which influenced individuals to fluctuate eating patterns and develop behaviors such as avoidant and restrictive food intake, further increasing the risk of eating disorders. Wade et al. [25] found that internalization of media appearance ideals were significantly and positively associated with eating disordered behaviors. In addition, some studies have also found that the communication of media ideal body image affects individuals’ emotional, behavioral, and physical health in China, such as the internalization of media appearance ideals affect individuals’ appearance anxiety, transitional control of weight and body size [26], formation of restrictive diet [27], and eating disorders [14]. In summary, the internalization of media appearance ideals has a significant impact on both individuals’ eating attitudes and behaviors. Therefore, the present study hypothesizes that internalization of media appearance ideals has a significant positive predictive effect on adolescent eating disorder tendency.

### The chain mediation of body esteem and body image disturbance

Most studies have shown that there is a significantly negative correlation between physical esteem and eating disorder tendencies, i.e., the higher the level of physical esteem, the lower the eating disorder tendencies [28, 29]. More importantly, adolescents are in a period of rapid development of self-awareness and their psychological “semi-infantile and semi-mature” performance makes this group more vulnerable to physical self-esteem [30, 31], which in turn affects individual eating disorder tendencies. The dimensional theory of self-evaluation also suggests that individuals determine the level of self-worth and self-esteem by evaluating their appearance, abilities, and personality [32]. And body esteem, as an important dimension of individual self-evaluation [33, 34], influences the individual’s perception and emotional experience of the self-body by self-evaluation, and thus influences the individual’s eating behavior. Thus decreasing of body esteem may be an important factor to the increased tendencies of eating disorders.

In addition, there is a negative relationship between the internalization of media appearance ideals and body esteem. Specifically, the easier it is to identify the ideal body presented by the media as one’s own ideal, the lower the individual’s body esteem. For example, Soohinda et al. [35] have found a negative relationship between the level of internalization of media appearance ideals and body esteem among female college students, while Ricciardelli et al. [36] have found this relationship among male adolescents. In addition, the negative relationship has also been verified in different cultures [37]. The above studies suggest that there is a negative impact of internalizing the media appearance ideals on body esteem. Sociocultural theory of body image suggest that the greater the difference between an individual’s perceived self-image and the ideal body image, the likely it is to lead to body dissatisfaction and low body self-esteem will be [17]. Low body esteem as a negative experience is often accompanied by body shame, rumination and negative social appearance evaluation fears and anxiety [38, 39], which in turn can compel individuals to take action to lose weight and maintain what they perceive as a “good” body shape, thus developing eating disorder tendencies. In addition, some studies have found that body esteem plays a mediates role in internalizing ideal body size and eating disorders [40], social media use and female eating disorder tendencies [38, 41], and body satisfaction and eating disorders [42, 43], respectively. Some studies on body esteem and eating disorder tendencies in adolescents currently focus on the field of physical exercise and the female college student in China [44], but few researches have been carried out on adolescent. Therefore, this study hypothesizes that there is a significant mediating effect of body esteem between internalization of media appearance ideals and eating disorder tendencies.

The tripartite influence model suggests [17] that the media is a key factor influencing the individuals body image, and that body image information presented in media may induce individuals to make social comparisons, which in turn triggers denial or even aversion to their own body image, resulting in body image disturbance [17, 45]. Additionally, social media is known to be highly appearance-focused, with content and messaging promoting idealized, unrealistic, and unachievable beauty ideals and standards, this directly aggravates the generation of body image disturbance. Passively using social media also had a significantly positive predictive effect on body image disturbance [46], and young women exposed to “thin is ideal” advertisements were more dissatisfied with their body image and reported experiencing more negative emotions. Thus, as a sociocultural transmission tool, when individuals internalize images presented in the media that are characterized as “thin” or “muscular”, internalization of media appearance ideals can lead to cognitive biases about their own body image, which in turn can cause body image disturbance.

Recent studies have shown that body image disturbance is one of the important predictors of eating disorder tendencies in adolescents. Body image disturbance include dissatisfaction with physical appearance, excessive attention to body image, and excessive concern about body shape, all of which may lead to the onset and development of eating disorders [47]. Some studies have found that body image disturbance is positively associated with eating disorder tendency. That is, the more severe the body image disturbance, the higher the tendency of eating disorders [10]. On the one hand, emotion regulation and avoidance theories of eating disorders suggest that individuals will relieve or escape from negative emotional experiences (such as worry and anxiety) through disordered eating behaviors [24]. On the other hand, the prevalence of body image disturbance is high among adolescents in China [16], and 30.5% of adolescents with body image disturbance have taken weight loss measures [48], so it is necessary to deepen the study of body image disturbance and eating disorder tendencies among adolescents. At the same time, body image disturbance may be a mediator between the internalization of media appearance ideals and eating disorder tendencies [49, 50]. Sociocultural theory suggests that the more adolescents are exposed to and identify with sociocultural messages about the ideal body image from society and the media, the likely they are to develop cognitive and perceptual biases towards their own body image, which can lead to worry and anxiety and irregular eating behaviors [48, 51]. Therefore, the present study hypothesizes that body image disturbance significantly mediates the relationship between the internalization of media appearance ideals and eating disorder tendencies.

Furthermore, there is a strong relationship between body esteem and body image disturbance. Increasing body esteem can reduce dissatisfaction and anxiety about one’s body image, thereby reducing levels of body image disturbance [38]. In addition, body esteem can promote more positive and healthy body perceptions and body shape perceptions, thus improving body satisfaction [52]. The cognitive self-assessment model suggests that individuals tend to evaluate their own appearance, which can be interpreted as a level of self-approval. When individuals do not adequately approve of themselves, they are likely to have a negative view on their body image and may perceive others to have negative views of them. As a result, their self-esteem levels will drop [53]. What’s more, the media plays a crucial role in shaping social appeal. The shaping of ideal image influences how adolescents perceive their body image [10, 54] and generates concerns about their body image when comparing the ideal image with the real image, which triggers emotional and psychological distress, leading to disordered eating [55, 56]. Therefore, the present study hypothesizes a significant chain mediating role of body esteem and body image disturbance in the internalization of media appearance ideals and eating disorder tendencies.

### The moderating role of social support

High levels of social support can weaken the relationship between various forms of social anxiety and eating disorder symptoms [57, 58], but inadequate or lack of social support can lead to the persistence of negative emotions, thereby increasing the occurrence of eating disorders, prolonging the cycle of eating disorders and exacerbating difficulties in recovery from bulimia [59]. The buffering effect of social support suggests that when individuals are under stress, the help and support from individuals social support system can reduce the negative effects of stress [60]. That is, social support facilitates the adjustment of individuals’ negative perceptions of eating behaviors, which in turn adjusts their eating behaviors. Previous empirical studies have also shown that perceived social support in early adolescence is negatively associated with negative eating attitudes and behaviors later in life [61].In summary, it is suggested that social support may help adolescents cope with frustration and prevent eating problems, as well as enhance personal well-being during key stages of adolescent development.

In addition, social support is a common moderating variable and has been found to play a moderating role in negative emotions or bad behaviors. For example, social support has been found to play a significant moderating role in the relationship between peer aggression and adolescents’ subjective well-being [62], and between narrative disorders and interpersonal relationships among secondary school students respectively [63]. Further, previous researches have shown that social support mitigates the relationship between social anxiety and eating disorders [57], protects individuals against bad eating behaviors (e.g., restrictive or binge eating behaviors) [64, 65]. And the protective effect on emotional and eating problems is stronger when the social support is higher [66]. It is evident that social support can act as a buffer and protector when individuals have adverse emotional feelings as well as problematic behaviors due to external stress. Therefore, this study hypothesizes that social support plays a significant moderating role between body image disturbance and eating disorder tendencies.

**Fig. 1:**
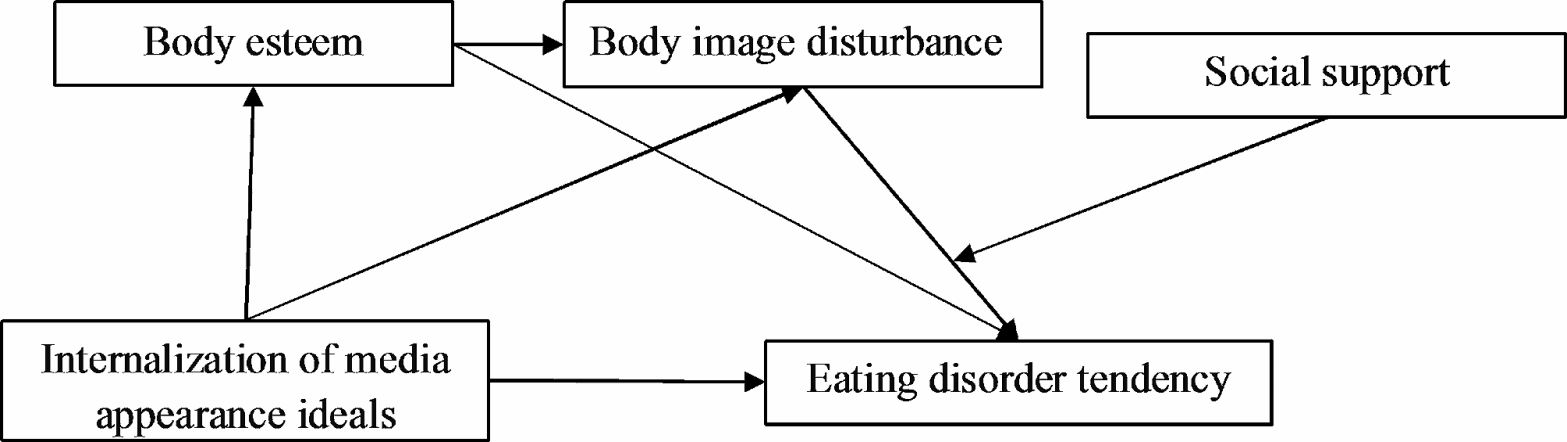
The hypothesize model

In summary, this study intends to investigate three main questions: (1) how internalization of media appearance ideals are relate to eating disorder tendencies; (2) whether body esteem and body image disturbance play a chain mediating role in the relationship between internalization of media appearance ideals and eating disorder tendencies; and (3) whether social support moderates the second half of this chain-mediated model (see Fig. 1).

## Materials and methods

### Participants

This study recruited 1523 middle and high school students in Guanzhou, China, to complete questionnaires. The mean age of the study participants were 14.65 years (SD = 1.81), with 728 (47.80%) males and 795 (52.20%) females. The number and the percentage of students in middle school are 340 (22.30%) in the grade, 285 (18.71%) in the second grade, and 137 (9.70%) in the third grade respectively. The number and the percentage of students in high school are 291 (19.71%) in the first grade, 207 (13.60%) the second grade and 263 (17.30%) in the third grade respectively. In addition, when screening for eating disorder tendencies, students scoring 0 to 10 are 1161 (76.23%), scoring 11 to 19 are 237 (15.56%), and scoring 20 or more are 125 (8.21%). The specific details are presented in Table 1.

**Table 1:** Demographic variables and screening results for eating disorder tendency of the study participants

Variables	Groups	Sample (*N* = 1523)	%
Gender	Male	728	47.80
	Female	795	52.20
Grade	Grade 7	340	22.30
	Grade 8	285	18.70
	Grade 9	137	9.70
	Grade 10	291	19.10
	Grade 11	207	13.60
	Grade 12	263	17.30
Monthly income^a^	<2000	47	3.10
	2000–5000	301	19.80
	5000–10,000	714	46.90
	10,000–20,000	309	20.30
	>20,000	152	10.00
ETA-26	0–10	1161	76.23
	11–19	237	15.56
	≥ 20	125	8.21

^a^ The monetary unit of income is Yuan; US$1 equals about 7.19 Yuan

### Measures

#### The sociocultural attitudes towards Appearance Questionnaire-3 (SATAQ-3)

To assess sociocultural attitudes towards appearance, participants completed the SATAQ-3 [67]. Liu et al. [68] revised the questionnaire into China, achieving satisfactory psychometric properties. The questionnaire consists of 15 items on two dimensions: media internalization and media concern. Responses are given on five-point scales with the anchor points 1 (completely disagree) to 5 (totally agree), with higher scores reflecting a stronger tendency to adapt appearance toward sociocultural expectations. In this study, the Cronbach’s alpha coefficient for the scale was 0.83, with 0.83, 0.67 for each dimension respectively.

#### Eating attitude Test-26 (EAT-26)

The ETA-26, developed by Garner et al. [69], was used to assess eating attitude. The questionnaire consists of 26 items on three dimensions: dieting, bulimia and food concern, and oral control, each of which is divided into six levels according to the severity of symptoms (1 = never, 6 = always). The study used the recommended scoring method for screening (3 = always, 2 = extremely often, 1 = often, 0 = sometimes/rarely/never). The higher the score, the more abnormal the eating attitude and the more likely the eating disorder is to occur. A score of 0–10 is approximately normal; a score of 11–19 has a tendency to be anorexic or bulimic; and a score of 20 or above is highly likely to be anorexic or bulimic. In this study, the Cronbach’s alpha coefficient for the scale was 0.83, with 0.82, 0.70 and 0.74 for each dimension respectively.

#### Physical self-description questionnaire (PSDQ)

To assess body esteem, the Marsh et al. [70] PSDQ was employed. Yang et al. [71] revised the questionnaire into China, achieving satisfactory psychometric properties. We used the overall body dimension of the scale to measure body esteem, with 6 items. Responses are given on six-point scales with the anchor points 1 (completely disagree) to 6 (completely agree), with higher scores reflecting higher levels of individual body esteem. In this study, the Cronbach’s alpha coefficient for the scale was 0.91.

#### Body image Depression Questionnaire (BIDQ)

The BIDQ developed by Gao et al. [72] was used to measure the level of body image disturbance of adolescents. The BIDQ consists of 25 items on four dimensions: body image disturbance, gender disturbance, sexual organ disturbance and appearance disturbance. The scale is scored on a 3-point scale (1 = agree, 3 = disagree), with lower scores indicating higher levels of body image disturbance. In this study, the Cronbach’s alpha coefficient for the scale was 0.88, with 0.80, 0.65, 0.60 and 0.87 for each dimension respectively.

#### Multidimensional scale of Perceived Social Support (MSPSS)

To assess social support, the Zimet et al. [73] MSPSS was employed. Zhao and Li [74] revised the questionnaire into China, achieving satisfactory psychometric properties. The MSPSS is a widely used 12-item measure that assesses perceived social support from three sources: friends, family, and significant others. Responses are given on five-point scales with the anchor points 1 (completely disagree) to 5 (totally agree), with higher scores reflecting higher levels of social support. In this study, the Cronbach’s alpha coefficient for the scale was 0.93, with 0.89, 0.86 and 0.86 for each dimension respectively.

### Procedure and analysis

This study used an anonymous self-report questionnaire, administered as a group in a class with a paper version of the questionnaire. Subjects were rigorously trained before administering the questionnaire and the administration process was carried out according to strict procedures. The collected data were analyzed using SPSS 26.0 for basic data organization, and AMOS 24.0 was used to complete the analysis of the mediation and moderation model. Pearson correlation was conducted to examine the correlations between all main variables. The primary analysis of the mediation model method was path analysis with maximum likelihood (ML) estimation. The bootstrap method, which repeatedly draws random samples from the original data with replacement, was used to evaluate the mediation effect. We used 5000 bootstrap resamples for this analysis to compute the 95% confidence intervals. Confidence intervals were then tested for significance by examining whether or not they contained zero. In addition, it has been considered that the gender, age, grade and monthly household income of the subjects may have an impact on the results and therefore were set as control variables in this study [75–77]. The study was approved by the Ethics Committee of Guanzhou University in accordance with the declaration of Helsinki. And all methods were carried out in accordance with the Declaration of Helsinki. All subjects and subjects’ parents gave written informed consent in accordance with the Declaration of Helsinki.

## Results

### Demographic statistics and correlations

In this study, the data were tested for common method variance (CMV) using *Harman’s Single-Factor Test*, and the first factor only accounted for 16.21% (less than 40% of the total variation), so there was no significant CMV exists.

The demographic characteristics and correlation of the research population are summarized in Table 2. The results found that internalization of media appearance ideals were negatively (*p* < 0.01) associated with body esteem, body image disturbance and social support, while it was positively (*p* < 0.01) associated with eating disorder tendencies.

**Table 2:** Descriptive statistics and correlations for the study variables (*N* = 1523)

Variables	M	SD	1	2	3	4	5
1 IMAI	5.14	1.31	1				
2 Body esteem	3.80	1.24	− 0.23^**^	1			
3 Body image disturbance	2.31	0.38	− 0.53^**^	0.42^**^	1		
4 Eating disorder tendency	0.29	0.33	0.29^**^	− 0.31^**^	− 0.43^**^	1	
5 Social support	4.95	1.35	− 0.05^*^	0.29^**^	0.20^**^	− 0.10^**^	1

*Notes. M* = mean, *SD* = standard deviation. IMAI = internalization of media appearance ideals. ^*^p < 0.05; ^**^p < 0.01

### The chain mediating effect

Using Model 6 in the SPSS macro program PROCESS (Model 6 is a chain mediation model) to test the mediating effect of body esteem and body image disturbance in the relationship between internalization of media appearance ideals and eating disorder tendencies under the control of gender, age, grade and income. The result showed that internalization of media appearance ideals positively predicted eating disorder tendencies (*β* = 0.33, *p* < 0.001); when both internalization of media appearance ideals and body esteem predicted body image disturbance, internalization of media appearance ideals significantly negatively predicted body image disturbance (*β*=-0.42, *p* < 0.001) and body esteem significantly positively predicted body image disturbance (*β* = 0.31, *p* < 0.001); when internalization of media appearance ideals, body esteem and body image disturbance simultaneously predicted eating disorder tendencies, internalization of media appearance ideals significantly positively predicted eating disorder tendencies (*β* = 0.13, *p* < 0.001), body esteem significantly negatively predicted eating disorder tendencies (*β*=-0.15, *p* < 0.001) and body image disturbance significantly positively predicted eating disorder tendencies (*β*=-0.35, *p* < 0.001). The results of mediating effect analysis (see Table 3) showed that body esteem and body image disturbance had a significant mediating effect between the internalization of media appearance ideals and eating disorder tendencies respectively, and the chain mediating effect was also significant.

**Table 3:** The chain mediation effect of body esteem and body image disturbance between internalization of media appearance ideals and eating disorder tendencies

Path	β	Effect Size	Bootstrap 95% CI
IIMIA→EDT	0.125	0.379	[0.072, 0.178]
IMIA→BE→EDT	0.034	0.103	[0.005, 0.012]
IMIA→BID→EDT	0.147	0.446	[0.020, 0.050]
IMIA→BE→BID→EDT	0.024	0.073	[0.116, 0.018]

*Notes.* IMAI = internalization of media appearance ideals, BE = body esteem, BID = body image disturbance, EDT = eating disorder tendency

### The moderated mediation test

**Fig. 2:**
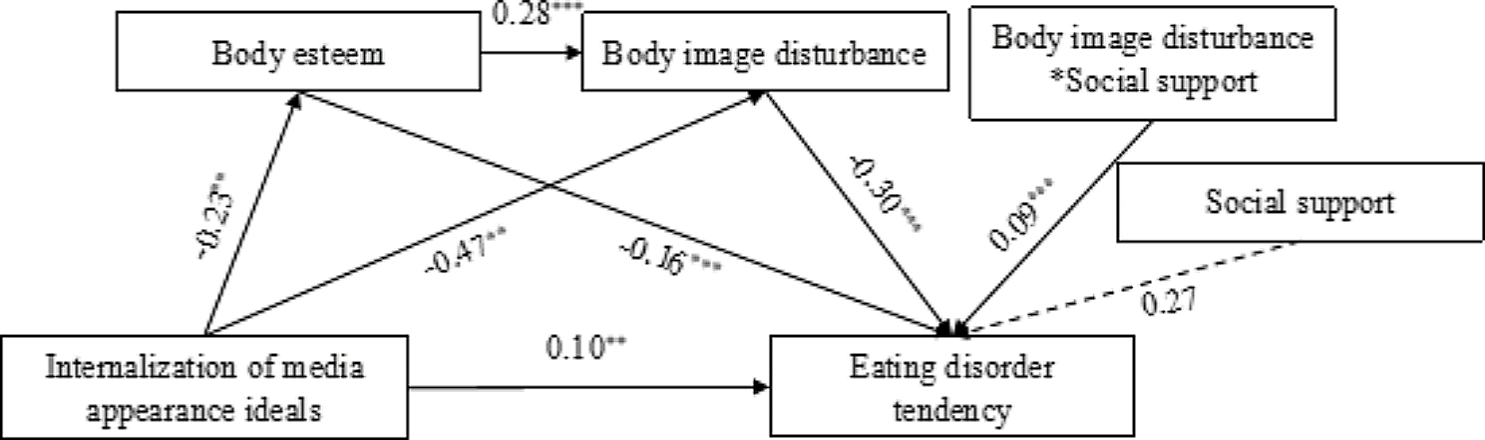
Moderating effect of social support. The lower the body image disturbance score, the higher the level of body image disturbance. **p < 0.01; ***p < 0.001

According to the hypothesize model (see Fig. 1) proposed in this study, we used AMOS 24.0 to empirically examine the moderated mediation effect. We explored whether social support moderates the relationship between body image disturbance and eating disorder tendencies. The model fits well (χ2/df = 5.293, RMSEA = 0.053, NFI = 0.979, IFI = 0.983, TLI = 0.966, CFI = 0.983). The standardized estimates and significance of each path coefficient are shown in Fig. 2.

The moderated mediated effects of body esteem and body image disturbance were both significant in this model, with the effect sizes of the two mediated pathways accounting for 12.88% and 47.80% of the total effect size respectively, and the chain mediated effects of body esteem and body image disturbance together were significant, with the chain mediated effect size accounting for 6.44% of the total effect size, as shown in Table 4.

**Table 4:** The chain mediation effect of body esteem and body image disturbance between internalization of media appearance ideals and eating disorder tendencies

Path	β	Effect Size	Bootstrap 95% CI
IIMIA→EDT	0.097	0.329	[0.048, 0.149]
IMIA→BE→EDT	0.038	0.129	[0.026, 0.051]
IMIA→BE→EDT	0.141	0.478	[0.118, 0.168]
IMIA→BE→BID→EDT	0.019	0.064	[0.015, 0.026]

*Notes.* IMAI = internalization of media appearance ideals, BE = body esteem, BID = body image disturbance, EDT = eating disorder tendency

**Fig. 3:**
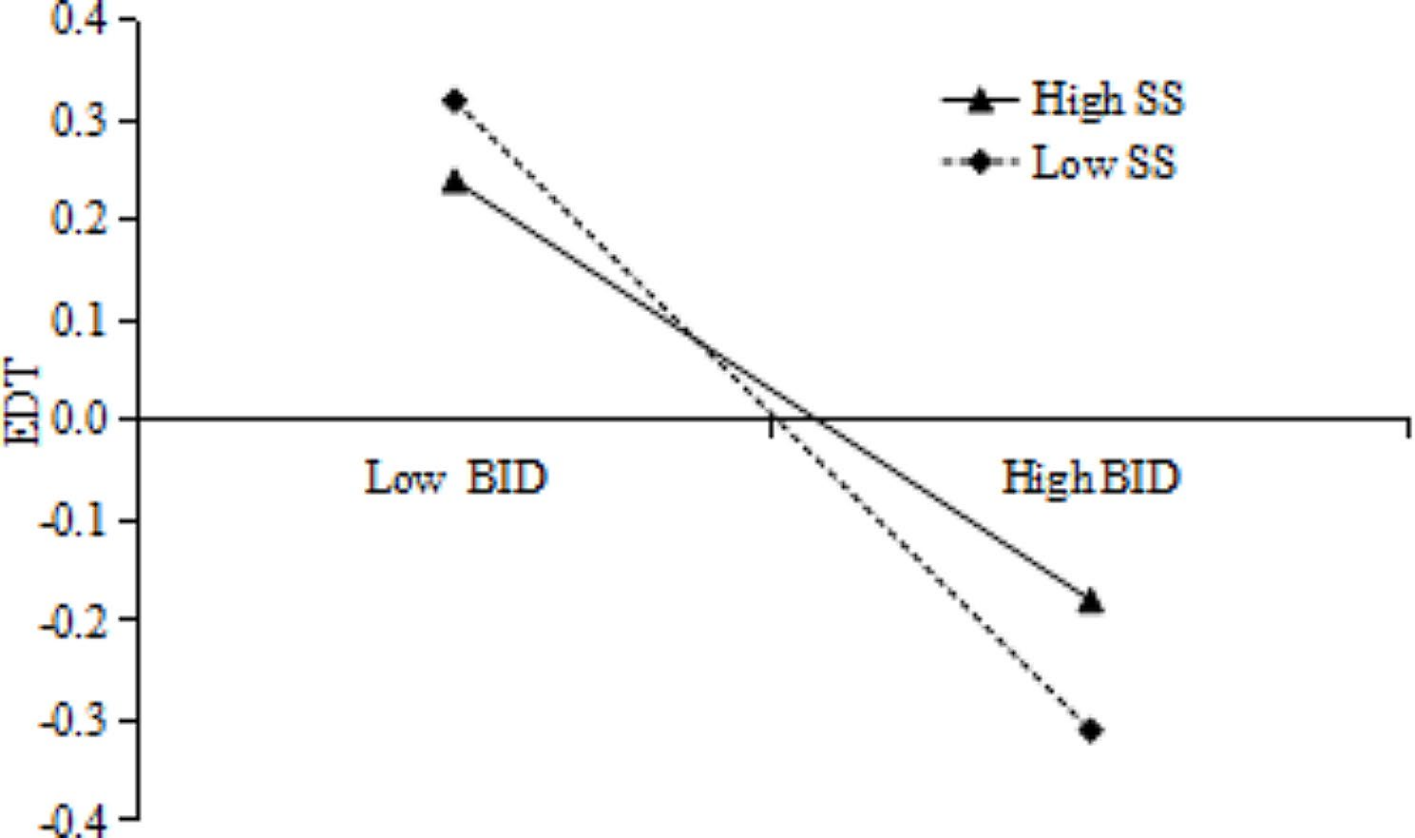
The interaction between body image disturbance and social support. BID = body image disturbance, SS = social support; EDT = eating disorder tendency

To illustrate whether social support moderated the correlation of body image disturbance with eating disorder tendencies, we plotted predicted eating disorder tendencies against higher or lower body image disturbance for social support (see Fig. 3). The results showed that body image disturbance was a significant negative predictor of eating disorder tendencies when moderated by low levels of social support (*β*=-0.38, *p* < 0.001, CI [-0.449, -0.303]); and a significant negative predictor of eating disorder tendencies when moderated by high levels of social support (*β*=-0.22, *p* < 0.01, CI [-0.307, -0.129]). This result suggested that when adolescents’ perceived level of social support was higher, the less negative effect of body image disturbance on eating disorder tendencies.

## Discussion

Eating disorders pose a serious threat to the physical and psychological health of adolescents as a global public health problem. In the screening test (EAT-26) of this study, although the majority of students were in the normal range (76.23%), more than one fifth (23.77%) had anorexic or bulimic tendencies and were at high risk of having anorexia or bulimia. It is shown that eating disorder tendencies are more common among adolescents, which cannot be ignored. The present study shows that internalization of media appearance ideals significantly and positively predicted eating disorder tendencies, which is consistent with previous research [78, 79]. When the perceived media pressure is internalized by individuals, self-comparisons will be triggered, with girls mainly comparing themselves with the internalized thin-ideal [38] and boys mainly comparing themselves with the internalized muscle-ideal [80]. Basing on this, cognitive and emotional imitation and identification are triggered [81], and may lead individuals to be overly concerned about their body and diet, thus developing extreme eating attitudes and behaviors that may further develop into eating disorders [82]. In addition, previous researches have found that images of thinness in the media may be a disinhibited factor for restrictive eaters. That is, exposing to these images can lead to opposite effects on restrictive eaters [83, 84], and the effects are negative in the long term. Internalization of media appearance ideals may act as a reminder cue for non-restricted eaters [81], e.i, the internalized image will trigger the eaters’ excessive attention, which may result in contraction bias and visual adaptation and the tendency to overeat. This is supported by cognitive behavioral theory [85], which suggests that key stimulus features (e.g. thin and muscular ideals) activate cognitive biases in individuals with high level self-schema [86], and that individuals may develop eating disorder tendencies in the processing.

This study has found that body esteem mediates the relationship between the internalization of media appearance ideals and eating disorder tendencies. It’s suggested that individuals with a greater degree of internalization of media images are likely to have lower body esteem that triggers a higher risk of eating disorders. This result further supports the view of the biopsychosocial model theory of body image [87]. That is, individuals who internalize the ideal appearance of thinness and muscularity conveyed by social media may develop negative evaluations and dissatisfaction, a psychological state that lowers an individual’s body esteem. When body esteem is lowered, individuals may adopt unhealthy eating behaviors (e.g. dieting, binge eating, etc.) in an attempt to change their body image, thus increasing eating disorder tendencies [41, 88]. In addition, according to sociocultural theory of body image, high sociocultural expectations of body image make individuals likely to experience low body esteem, triggering negative emotions [17]. Individuals with negative emotions are often led to diet and other weight loss behaviors, which in turn triggers eating disorder tendencies [89]. Our findings suggest that to prevent adolescents from eating disorders, we need to focus on the ideal body image and body esteem, and help adolescents develop healthy self-esteem and self-evaluation.

In addition, the present study explored the role of body image disturbance in the relationship between the internalization of media appearance ideals and eating disorder tendencies and has found that body image disturbance plays a significant mediating role, which is consistent with previous researches [38, 90]. The media makes individuals actively or passively exposed to the ideal body image, and such contact may lead to adolescents pay attention to or pursuit the ideal body type [17]. Furthermore, adolescents are at an important time in the development of dramatic body changes, where individuals are more attentive to body messages and therefore more vulnerable to body image disturbance [88, 91]. The combination of media exposure and selective attention leads to individuals’ dissatisfaction with their own bodies, which triggers body image disturbance and further increases individuals’ risk of eating disorders.

Moreover, this study has also found that body esteem and body image disturbance chain mediate between the internalization of media appearance ideal and eating disorder tendencies. It’s suggested that the internalization of media appearance ideal increases individuals’ eating disorder tendencies by reducing their body esteem and increasing body image disturbance. Body image theory suggests that an individual’s the self-esteem level is a major factor to form the self-concept of body image [92–94]. Meanwhile, the cognitive self-assessment model suggests that individuals tend to evaluate their own appearance to achieve self-approval [53], and that when individuals do not adequately recognize and when individuals do not fully recognize and accept their appearance, they are likely to develop a low-levels of body esteem and lead to body image disturbance [95]. So, the existence of this chain mediating effect should be noted to mitigate adolescents’ eating disorder tendencies by strengthening body esteem and reducing body image disturbance. At the same time, there is also a need to pay attention to the influence of the media environment on adolescents’ body image and improve adolescents’ cognitive and critical ability for the ideal body image.

The results of this study shows that social support moderating the relationship between body image disturbance and eating disorder tendency, and that higher social support reducing the negative effect of body image disturbance on eating disorder tendencies. It’s suggested that as an important protective factor, social support buffers the negative emotions generated by media stress, reducing the risk of eating disorders [15]. And this is consistent with the social buffering effect model and previous researches [96]. Social support helps individuals to reassess potential threats, provide positive emotions [97], and offer information and substantial help [98], and it helps to reduce the individual’s identification with thinness and muscularity as the ideal body and increasing feelings of self-worth [99], increase the individual’s social engagement and social support network [100], thus alleviating the negative emotions of body disturbance. In addition, cognitive resource theory suggests that individuals need to consume cognitive resources when faced with stress, and that excessively-consumed cognitive resources may lead to the onset and intensification of stress reactions [101]. Social support can provide additional cognitive resources to help individuals cope with the negative emotions associated with internalization of media appearance ideals. Specifically, social support can alleviate the emotional burden that individuals develop in response to the internalization of media appearance ideal and release additional cognitive resources. Thus, social support can help alleviate physical disturbance and mitigate the onset and intensification of eating disorder tendencies.

### Implications

This study reveals the mechanism by which the internalization of media appearance ideals affects the eating disorder tendencies among adolescents, and provides empirical support and theoretical guidance for the prevention and intervention of eating disorder problems. Firstly, there should be strict supervision over the media to guide students to view the messages about the ideal body image correctly. Of course it is not enough only to restrain external factors. Schools and parents should guide students to use the internet correctly and to view the ideal body image in the media rationally, so that these over-glorified and unhealthy body images will not affect the correct perception. Secondly, adolescents should raise their level of body self-esteem and improve their sense of body self-worth. Adolescents are at the stage of forming their worldview and values. And the reduction of extreme eating behaviors requires both parents, schools and teachers to guide students to view and accept their bodies correctly and to develop a correct concept of body beauty. Thirdly, we should emphasize with body image education for adolescents. Body image education focuses on individuals’ cognitive, emotional and behaviors responses to their own bodies. In secondary school, adolescents are at a critical stage of physical and psychological development and are faced with many challenges [102]. Therefore, it is important to enhance body image education for adolescents. Fourthly, we should give support in many ways, while improving the individual’s ability to perceive social support. Therefore, families and schools should pay more attention to the emotional state and behaviors of adolescents, and give full play to the power of peer support to help each other, so that adolescents can have more support and be more positive when dealing with external negative influences and their own negative emotional experiences. In addition, this study did not focus on positive body image and digital media literacy. Future research should consider incorporating the concepts of positive body image and digital media literacy into the study. By considering these factors, researchers can gain a more comprehensive understanding of the complexity surrounding body image among Chinese adolescents and provide potential intervention and support strategies.

## Conclusions

The present study yielded the following conclusions (1) Internalization of media appearance ideals significantly and positively predicts adolescents’ eating disorder tendencies; (2) Internalization of media appearance ideals significantly predicts adolescents’ eating disorder tendencies through the mediating effects of body self-esteem and body image disturbance, as well as through the chain mediated effects of body self-esteem and body image disturbance; (3) And social support plays a moderating role between body image disturbance and eating disorder tendencies, with higher social support reducing the negative effect of body image disturbance on eating disorder tendencies.

## Data Availability

The datasets generated and analyzed during the current study are not publicly available due to confidentiality and privacy related issues but are available from the corresponding author on reasonable request.
